# A Possible Role for Singlet Oxygen in the Degradation of Various Antioxidants. A Meta-Analysis and Review of Literature Data

**DOI:** 10.3390/antiox7030035

**Published:** 2018-02-27

**Authors:** Athinoula L. Petrou, Petros L. Petrou, Theodoros Ntanos, Antonis Liapis

**Affiliations:** 1 Laboratory of Inorganic Chemistry, Department of Chemistry, National and Kapodistrian University of Athens, Panepistimiopolis, 15771 Athens, Greece; theontanos@hotmail.com (T.N.); a-liapis@hotmail.com (A.L.); 2 Private practice in Cardiology, 6–8 Acheans Street, Nicosia 1101, Cyprus; lambrosp@spidernet.com.cy

**Keywords:** antioxidants, oxidative stress, singlet oxygen, free energy of activation, enthalpy of activation, entropy of activation, activation energy

## Abstract

The thermodynamic parameters E_act_, ΔH^≠^, ΔS^≠^, and ΔG^≠^ for various processes involving antioxidants were calculated using literature kinetic data (k, T). The ΔG^≠^ values of the antioxidants’ processes vary in the range 91.27–116.46 kJmol^−1^ at 310 K. The similarity of the ΔG^≠^ values (for all of the antioxidants studied) is supported to be an indication that a common mechanism in the above antioxidant processes may be taking place. A value of about 10–30 kJmol^−1^ is the activation energy for the diffusion of reactants depending on the reaction and the medium. The energy 92 kJmol^−1^ is needed for the excitation of O_2_ from the ground to the first excited state (^1^Δ_g_, singlet oxygen). We suggest the same role of the oxidative stress and specifically of singlet oxygen to the processes of antioxidants as in the processes of proteinaceous diseases. We therefore suggest a competition between the various antioxidants and the proteins of proteinaceous diseases in capturing singlet oxygen’s empty π* orbital. The concentration of the antioxidants could be a crucial factor for the competition. Also, the structures of the antioxidant molecules play a significant role since the various structures have a different number of regions of high electron density.

## 1. Introduction

In a recent article of ours [1], we have presented a possible role of oxidative stress and singlet oxygen in diverse diseases. In that article we have presented the calculated values of the free energies of activation of various reactions of proteins of proteinaceous diseases. From the values of the ΔGs^≠^, we came to the conclusion that the rate determining steps of the various reactions that were studied could be the formation of singlet oxygen from ground state oxygen and the diffusion of the various reactants, proteins, DNA, in general biomolecules. 

Having presented the above (possible) role of singlet oxygen (a major component of oxidative stress) in the pathogenesis of neurodegenerative and other disorders, we concluded that antioxidant administration or donation of reagents bearing regions of high electron density (bases) may be useful in the prevention and treatment of neurodegenerative diseases and the other disorders that were studied. In the last paragraph of [1] entitled “future strategies”, we have explained our goal for our studies that were to be followed. 

There we stated that to achieve the delay of disease progression, the candidate antioxidant or base must be supplied as early as possible, in order to prevent irreversible neuronal loss. The antioxidant or base should also be tailored to the region of generation of O_2_ (^1^Δ_g_), i.e., to neurons rich in lipids and/or metal ions. A critical property of the chosen antioxidant or basic properties-bearing reagent is to be able to penetrate the blood–brain barrier after systemic administration in order to reach a critical therapeutic level within the CNS (Central Nervous System). The antioxidants should be ones that could prevent lipid, protein and DNA peroxidation, and thus prevent production of the ^1^O_2_ (^1^Δ_g_). They could also be able to capture singlet oxygen’s empty π* orbital [1]. Suitable bases should also be ones that could donate a pair of electrons in the empty π* orbital of the produced O_2_ (^1^Δ_g_), in order to prevent its reaction with protein, lipid, DNA, etc. The antioxidants and bases (reagents bearing regions of high electron density) should react with the O_2_ (^1^Δ_g_), faster than lipids, proteins, DNA, etc. Thus, the ΔGs^≠^ of their reactions should be smaller than the ΔGs^≠^ that are presented in [1]. This means that the rate constants of the reactions should be greater than the ones reported in Supplementary Table S1 of [1].

In this paper, we present the results of our calculations on the activation parameters of reactions of various antioxidants with oxygen under various temperatures of treatment and we show that there is a possibility the reactive species in these oxidations to be the singlet oxygen species due to the values of the ΔGs^≠^. So, we suggest the same mechanism, that is, the formation of singlet oxygen and its reaction with the antioxidants. Concluding, we suggest that a competition might exist between antioxidants and the proteins of proteinaceous diseases in capturing the singlet oxygen’s empty π* orbital. 

Oxidative Stress, Reactive Oxygen Species (ROS), Proteinaceous diseases and the proteins related to them: Aβ-amyloid (Alzheimer’s disease, AD), Tau proteins [Alzheimer’s disease and Parkinson’s disease], α-Synuclein (Parkinson’s disease), Prion: PrP (Prion Proteins), Amylin or islet amyloid polypeptide (IAPP) (type II diabetes), α-Crystallins (Cataracts) are briefly presented in [1]. 

### 1.1. Antioxidants

Living organisms have complex systems of antioxidants in body tissues, in order to balance their oxidative state. These antioxidants are produced internally (endogenous) or are taken with diet (exogenous):

(a) Endogenous antioxidants—the ones which are produced by the body. Examples are: superoxide dismutase, catalase, glutathione peroxidase, and glutathione.
(b) Exogenous antioxidants—the ones which come from the diet. Examples are: vitamin C, carotenoids, polyphenols, sulforafanes, curcumin, anthocyanins, etc.

Many antioxidant-based clinical trials and therapeutic interventions have been disappointing in their therapeutic benefit [2]. It has been reported [3,4,5] that while numerous evidence points to increased levels of oxidative stress playing a causal role in a number of neurodegenerative conditions, the understanding of the specific role of oxidative stress in the genesis and/or propagation of neurodegenerative diseases is poorly defined. Even more challenging to the “oxidative stress theory of neurodegeneration” is the fact that many antioxidant-based clinical trials [3,4] and therapeutic interventions have been largely disappointing in their therapeutic benefit [2]. Based on our research work, we attribute the above finding to (a) the antioxidants that were tried might not be able to pass through the Blood Brain Barrier (BBB); (b) their structure might not be the suitable one to capture the empty π* orbital of O_2_ (^1^Δ_g_) bearing very few regions of high electron density that (c) their in vitro concentration might not be high enough in order to compete with the large amount of proteins present. We explain a, b, c, in more detail, below.

### 1.2. The Electronic Structure and the Energy above Ground State of Two Excited States of Oxygen. The First Excited State, the Singlet Oxygen State

The two outer electrons of molecular oxygen are located at the antibonding π* orbitals. In the ground state the two π* electrons occupy the equivalent in energy π*_2py_, π*_2pz_ orbitals (see below), whereas in the first excited state, the ^1^Δ_g_, they occupy one of these orbitals, leaving the second one empty. The highest occupied molecular orbitals (HOMO) of the ground state (^3^Σ_g_^−^), the first excited state (^1^Δ_g_) and the second excited state (^1^Σ_g_^+^) of the oxygen molecule are [6]:
π* ↑  ↑  π*π* ↑↓
  π*π* ↑  ↓  π*Ground stateFirst excited state Second excited state2S + 1 = 32S + 1 = 12S + 1 = 1^3^Σ_g_^−^^1^Δ_g_, 92 kJmol^−1^ above ground state ^1^Σ_g_^+^, 155 kJmol^−1^ above ground state

Thus, the first excited state, the ^1^Δ_g_ state, which is energetically 92 kJmol^−1^ above the ground state, bears an empty π* orbital where it can accommodate a pair of electrons. This availability of accepting a pair of electrons in the empty π* orbital, gives to singlet oxygen strong acidic properties. So, singlet oxygen having this ability can behave like a strong electrophile reacting with reagents bearing regions of high electron density, causing oxidative damage to them. These reagents may be biomolecules [1] or components of foods that are bearing antioxidant qualities [7]. Guanine, in the molecule of DNA, nitrogen and oxygen in proteinic molecules, nitrogen, and oxygen in amino acids, etc., can act as bases accommodating their pair of electrons in the singlet oxygen’s empty π* orbital [1]. Also antioxidants bearing electron densities like beta-carotene’s and lycopene’s conjugated double bonds, astaxanthin’s polyene chain, and terminal rings, sulforafane’s O, N, S lone pairs of electrons etc. can accommodate them in the empty π* orbital of singlet oxygen, provided that their geometry and energy suits to the π* orbital’s geometry and energy (see below). 

The general reactions taking place could be presented by the Equations:

$$\begin{array}{l} O_{2}\left({}^{1}\Delta_{g}\right)\;+\;\mathrm{biomolecule}\left(s\right)\;\mathrm{bearing}\;\mathrm{regions}\;\mathrm{of}\;\mathrm{high}\;\mathrm{electron}\;\mathrm{density}\;\overset{}{\underset{}{⇄}}\;\\ {\left[\mathrm{adduct}\;(O_{2}/\mathrm{biomolecule}\left(s\right))\right]}^{\neq }\overset{}{\underset{}{⇄}}\mathrm{oxidation}\;\mathrm{products}\;\mathrm{of}\;\mathrm{biomolecule}\left(s\right) \end{array}$$

$$O_{2}\left({}^{1}\Delta_{g}\right)\;+\;\mathrm{antioxidant}\overset{}{\underset{}{⇄}}{\left[\mathrm{adduct}\;\left(O_{2}/\mathrm{antioxidant}\right)\right]}^{\neq }\overset{}{\underset{}{⇄}}\mathrm{oxidation}\;\mathrm{products}\;\mathrm{of}\;\mathrm{antioxidants}$$

Singlet oxygen quenching [8] may be both physical and chemical quenching. Physical quenching leads to the deactivation of singlet oxygen by going to the ground triplet oxygen state. This can be achieved by energy transfer or charge transfer. There is no consumption of oxygen and no product formation. Singlet oxygen quenching by energy transfer takes place when the energy level of a quencher is very near or below that of singlet oxygen. Singlet oxygen quenchers by energy transfer are carotenoids with 9 or more conjugated double bonds. Tocopherols, curcumin, carotenoids, urate, phenolics, and ascorbate can quench singlet oxygen and the singlet oxygen quenching activity of carotenoids depends on the number of conjugated double bonds in their structure and the nature of substituents in the β-ionone ring. When a quencher bears high reduction potential and low triplet energy, it quenches singlet oxygen by a charge transfer mechanism. Quenchers of this type are amines, phenols (including tocopherols), iodide, sulfides, and azides, which all have many electrons. By donating an electron to singlet oxygen, the quencher forms a singlet state charge transfer complex and then by intersystem crossing changes the complex to the triplet state. Chemical quenching of singlet oxygen is a reaction involving the oxidation of a quencher. β-carotene, ascorbic acid, amino acids (such as histidine, cysteine, tryptophan, and methionine), peptides, tocopherols, and phenolics are chemical quenchers and they are all oxidized by singlet oxygen [9,10,11,12]. 

It should also be mentioned that many of the antioxidants that are discussed in this article quench singlet oxygen via both ways, physical as well as chemical quenching, and the physical quenching will not necessarily lead to destruction of the antioxidants. For example, carotenoids quench singlet oxygen mainly via energy transfer to generate the carotenoid triplet excited state, and this can then simply lose its energy as heat to decay back to the ground state. Tocopherols have also been shown to both chemically and physically quench singlet oxygen and astaxanthin is a very fast quencher of singlet oxygen.

Singlet Oxygen has also been extensively studied in relation to the photodynamic field [13,14,15,16,17].

### 1.3. Diffusion—Controlled Reactions, Activation Energy of Diffusion

Diffusion-controlled reactions (in solution) are reactions in which every collision of the reacting molecules leads to products. Their rates are only limited by the activation energy of diffusion that has values ranging from 10 to 30 kJmol^−1^ and are dependent on the nature of the solvent [18,19,20].

### 1.4. Food Antioxidants

**Figure 1:**
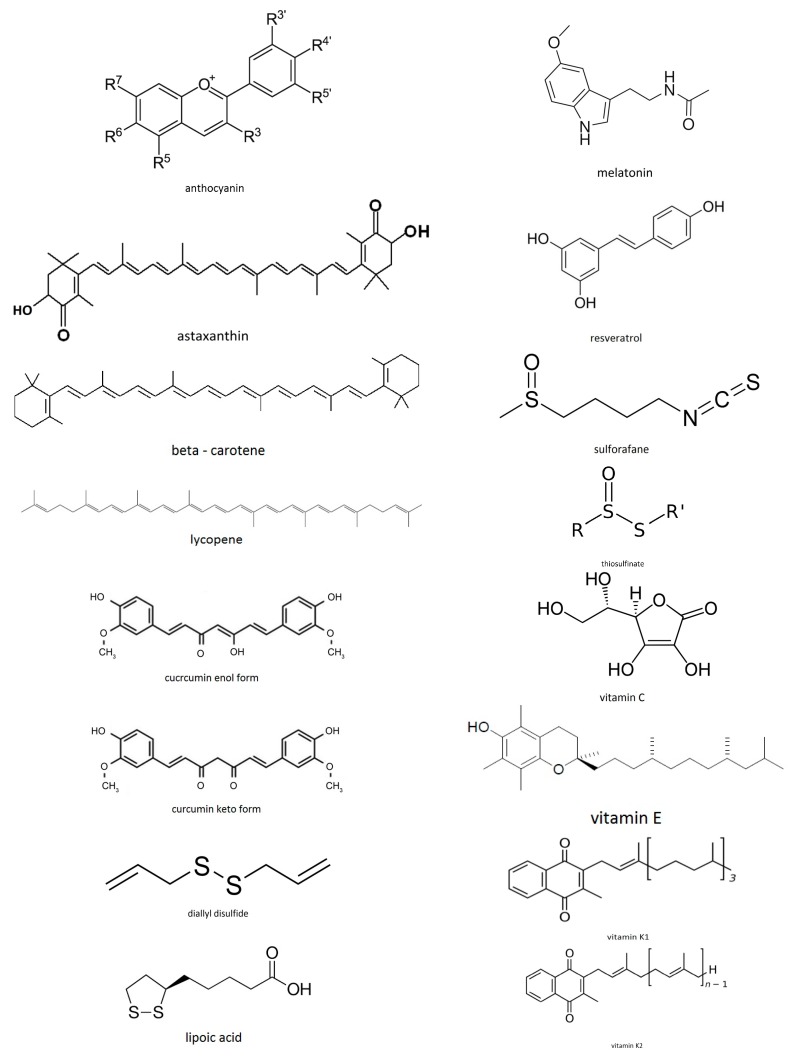

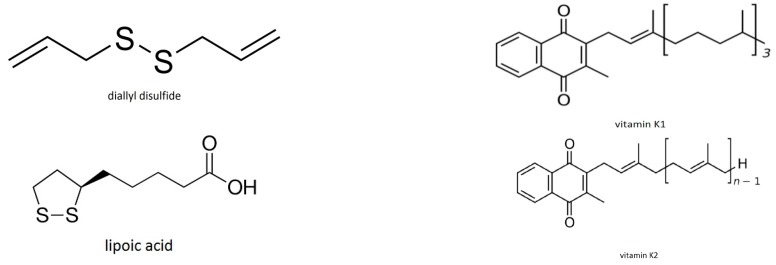
Chemical Structures of various antioxidants.

In the following presentation, the term “antioxidant” refers mainly to compounds in foods, such as anthocyanins, carotenoids, polyphenols, sulforafane, vitamins, etc. Also, in the description of the various antioxidants that follows, we will explain which regions of high electron density, or which pairs of electrons in their molecular structure can be accommodated to singlet oxygen’s empty π* orbital. We must note here that not only lone pairs must be available in the various compounds-candidates for reaction with singlet oxygen’s empty π* orbital, but also the energy of their molecular orbitals should suit the energy of the singlet oxygen’s (LUMO) orbitals. Overlapping between orbitals takes place provided that the energy and geometry of the orbitals suit. In Figure 1, structures of the antioxidants studied in the articles reviewed and others are presented.

### 1.5. Oxidative Stress, Singlet Oxygen and Antioxidants—The Goal of Our Research (Meta-Analysis and Review of Literature Data)

We have already presented evidence, using the Free Energy of activation values, that oxidative stress and specifically singlet oxygen play a significant role in neurodegenerative diseases, in cataract formation, in diabetes, and in carcinogenesis [1]. The brain is especially vulnerable to oxidative stress damage because of its high rate of oxygen consumption, its abundant lipid content, and the relative paucity of antioxidant enzymes as compared with other organs [1]. 

Reactive oxygen and nitrogen species, such as super oxide anion, hydrogen peroxide, hydroxyl radical, and nitric oxide (O_2_^–^•, H_2_O_2_, •OH, OONO^–^), and their biological metabolites also play an important role in carcinogenesis. The presence of free radicals in a biosystem could lead to mutation, transformation, and ultimately cancer [1 and refs therein].

Since oxidative stress, as it was outlined in [1] plays a crucial role in the above mentioned disorders (neurodegenerative disorders, diabetes, cataract, cancer) we searched for the Free Energies of activation that are needed in the biological (human) systems in order for the above various (proteinaceous) disorders to be initiated. A search in the literature led us to realize that the calculation of ΔG^≠^ [ΔG^≠^ = ΔH^≠^ − T·ΔS^≠^] of the various processes would give us reasonable results. Having done this, we came to the conclusion that the processes that we have studied, which lead to the above disorders, all have ΔG^≠^ values that vary between 92.8 and 127 kJmol^−1^ at 310 K [1]. Knowing that ~92 kJmol^−1^ is the energy needed for the production of singlet oxygen ([1] and refs therein), and that the activation energy of diffusion has values ranging from 10 to 30 kJmol^−1^, we were guided to propose a possible mechanism for the pathway(s) that leads to the studied disorders. The Free Energies of activation that were calculated using the data of the papers cited were similar. This similarity led us to conclude that a common mechanism is possibly taking place. The possible mechanism has been presented [1]. Having presented the above (possible) role of singlet oxygen (a major component of oxidative stress) in the pathogenesis of neurodegenerative and other disorders, we concluded that antioxidant administration or donation of reagents bearing regions of high electron density (bases) may be useful in the prevention and treatment of neurodegenerative diseases and the other disorders that were studied.

**Figure 2:**
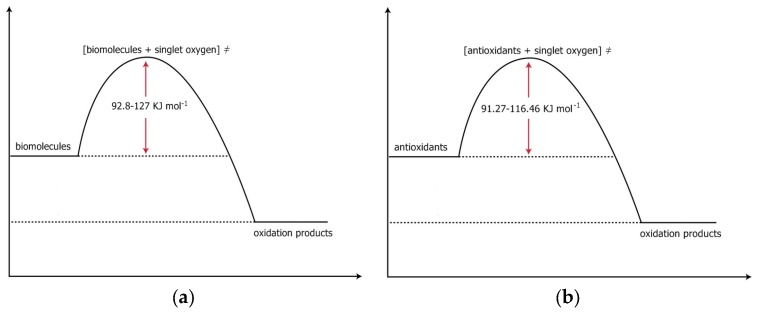
Free energies of activation for the reactions: (**a**) biomolecules + singlet oxygen and (**b**) antioxidants + singlet oxygen.

In this paper, we present the results of our calculations on the activation parameters of reactions of various antioxidants with oxygen under various temperatures of treatment and we show, based on the ΔG^≠^ values, that the reactive species in these oxidations might also be the singlet oxygen species, ^1^Δ_g_. Suggesting a mechanism for the reaction: antioxidant + O_2_, we propose the same rate determining step, that is, the formation of singlet oxygen that then reacts with the antioxidant. 92 kJmol^−1^ is the energy needed for the production of ^1^Δ_g_ and 10–30 kJmol^−1^ is the diffusion of the reactants’ activation energy. Having found similar values of ΔGs^≠^ for the reactions: antioxidants + O_2_ and proteins + O_2_ or biomolecules + O_2_ we suggest that a competition exists between antioxidants and the proteins of proteinaceous diseases or biomolecules in general in capturing the singlet oxygen’s empty π* orbital. In Figure 2, we present schematically the calculated values of ΔGs^≠^.

We also, in this paper, present and describe the characteristics of the structures of the various antioxidants that make them strong competitors with proteins, DNA, biomolecules in general that are candidates for capturing the singlet oxygen’s empty π* orbital.

The results of our calculations are presented in Table 1.

## 2. Method of Calculations

*The Eyring-Polanyi or activated complex theory relation—Free energy of activation*. For the calculation of the thermodynamic parameters of activation of the various processes that were studied and are presented in this article, we have applied the kinetic equations Arrhenius and Eyring-Polanyi according to the activated complex theory to the literature data (k, T) for the various antioxidant processess. The Arrhenius equation, the calculation of E_act_ from a plot of it, the Eyring-Polanyi equation, the calculation of ΔH^≠^ and ΔS^≠^ from a plot of it, and the calculation of ΔG^≠^ from the thermodynamic relation ΔG^≠^ = ΔH^≠^ − TΔS^≠^ are in detail presented in [1]. 

So, this paper is a meta-analysis and review of literature data that refer to antioxidant reactions with oxygen at various conditions (various temperatures, various pH, in the presence of light or without the presence of light, etc.). 

The ΔS^≠^ values for all of the antioxidants’ reactions with oxygen are found to be negative, implying that the reacting species (^3^O_2_) becomes more organised at the activated step, (^1^O_2_) justifying our mechanism (see below) [18,20,21,22].

## 3. Results

### Application of the Arrhenius and the Eyring-Polanyi Equations to Cases Related to Thermal and with Oxygen Treatment of Various Food Antioxidants 

We have applied the Arrhenius and the Eyring-Polanyi equations to literature data (k, T) that are related to thermal and with oxygen treatment of various food antioxidants and we have calculated the parameters E_act_, ΔH^≠^, ΔS^≠^, and ΔG^≠^. It has been shown that similar processes have similar ΔG^≠^ values (at the same temperatures), but may differ in the enthalpic and the entropic contributions [18,19,20,21,22]. We will present and compare the calculated activation parameters (ΔH^≠^, ΔS^≠^, ΔG^≠^, along with the E_act_) for the cases related to treatment of various food antioxidants (Table 1).

## 4. Discussion

From Table 1, it is shown that the values of E_act_ lie between 4.13 and 100.48 kJmol^−1^. The ΔH^≠^ values range between 1.13 and 97.75 kJmol^−1^. The ΔS^≠^ has values ranging between −318.66 and −46.68 JK^−1^ mol^−1^. 

The ΔG^≠^ values range between 91.27 and 116.46 kJmol^−1^ at 310 K for all of the cases of the food antioxidants studied. 

From the above ranges it is concluded that independently of the nature of the antioxidant (anthocyanin, beta-carotene, curcumin, lycopene, polyphenols, sulforafane, vitamin C, etc.) or the kind of process that is taking place, (thermal treatment, pH effect, with presence or without the presence of light, oxygen reaction, etc.), the values of E_act_, ΔH^≠^ and ΔS^≠^ cover a broad range, whereas the ΔG^≠^ values cover a narrow range. In this narrow range a value of 10–30 kJmol^−1^ is needed for the diffusion of the reactants, depending on the reaction and the medium. So, it remains a value of 92 kJmol^−1^. This value of 92 kJmol^−1^ is equal to the energy needed for the oxygen molecule, O_2_, to go from the ground state to the first excited state, the ^1^Δ_g_ state, the singlet oxygen state (see above Section 1.2). 

Concluding, we can say that, independently of the nature of the antioxidant and of the process that is taking place, the Free Energy of activation is actually equal to the energy that is needed for the production of singlet oxygen from ground state oxygen, [rate determining step (rds), excitation energy], to which the amount of the activation energy for the diffusion of the reactants is added. This fact raises some questions that need to be answered (see below, Section 4.3).

In the following analysis, we will, in short, present the various antioxidants, explaining specifically their ability, due to their structure, of capturing the singlet oxygen’s empty π* orbital. 

Before analyzing the various cases of antioxidants, we present the thermal injury due to normal body temperature [23]. After completing the analysis of all the cases of antioxidants that were studied, we present a case of oxygen-free reaction of diallyl disulfide for comparison with the reaction of diallyl disulfide in the presence of oxygen. We show that without the presence of O_2_, that is, without the formation of ^1^Δ_g_ the Free Energy of activation differs considerably from the values that were found with the antioxidants (diallyl disulfide) in the presence of O2 [135.81 kJmol^−1^ at 310 K vs. 101.21 kJmol^−1^ and 91.27 kJmol^−1^ at 310 K (case j), Table 1].

### 4.1. Analytically the Various Cases that were Studied Are: 

#### 4.1.1. Destructive Processes due to Normal Body Temperature

**Case a: Thermal injury due to normal body temperature [23]**

In this work, the authors report that at physiologic temperature, 37 °C, over 0.2% of cells are irreversibly lost from the proliferating population each hour as a result of heat injury [23]. The very important question raised by the authors is whether any of the destructive processes takes place at rates that are significant when compared to the lifespans of the cells and the various organisms. If so, then the heat destruction due to the physiologic temperatures must be considered as a critical factor determining the aging of organisms. The authors have found evidence that in the case of hamster cells in vitro this is true [23]. According to the authors the exact nature of the thermal destruction due to physiologic temperatures was not known (at 1973), but “*it probably involves the denaturation of certain proteins and nucleic acids. In simplest terms, thermal injury is a result of a number of molecular events, each of which is activated by the thermal free-energy pool* [23]”. The rate determining step (rds) has a rate constant which is the k of the slowest and critical reaction, if reactions of various rates are involved. The authors having found E_act_ = 8.0 electron volts/cell reported that this is equivalent to the rupture of only 30–40 hydrogen bonds/cell. They report ΔG^≠^, the free energy of activation as being 1.2 eV/cell. They also report ΔS^≠^ = 507 entropy units (eu). The above values are very high for most of the chemical reactions, but are typical of the denaturation of proteins, because of the required rupture of many hydrogen bonds [23]. This rupture of hydrogen bonds must be related to the rds since it leads to positive ΔS^≠^ (increase of degrees of freedom). In the cases that will follow, of the reactions of antioxidants, the ΔS^≠^ are negative suggesting loss of entropy, that is, increased organisation (decrease of degrees of freedom, decrease of the number of free species.). This will be explained by the mechanism for the rest of the cases, which will be presented, that is, the capture of the singlet oxygen’s empty π* orbital by the antioxidants’ lone pairs of electrons or regions of high electron density (unsaturated bonds) etc. If heat injury at 37 °C in mammalian cells in vitro proceeds at the rate indicated by the authors’ experiments, we could wonder about the accumulation of irreversible thermal lesions in long-lived cells in vivo. The authors are questioning whether this might be a major factor in the functional decline of the aging organisms. 

Our calculations according to the authors’ results give ΔG^≠^ = 112.8 (92 + 20.8) kJmol^−1^ at 310 K. We suggest a mechanism according to which singlet oxygen’s empty π* orbital is captured by the lone pairs of electrons of the –O and/or –N groups that participated in the hydrogen bonding. The hydrogen bonds were destroyed, as the authors suggest, by heat. The process is composite due to high values of ΔH^≠^ (766.89 kJmol^−1^) and ΔS^≠^ (2110.41 JK^−1^mol^−1^) (Table 1). Thus the ΔS^≠^ of the composite process is a sum of a negative value due to the reaction of singlet oxygen with proteins (^1^Δ_g_ + proteins) and a very positive value (ΔS^0^ > 0) due to the disruption of the hydrogen bonds that preceded. We thus suggest the establishment of pre-equilibria (prior to the rds) that have high positive values of ΔH^0^ and ΔS^0^. The 112.8 kJmol^−1^ at 310 K value shows that the denaturation of proteins and nucleic acids may be indeed a reaction with singlet oxygen’s empty π* orbital.

#### 4.1.2. Anthocyanins (Also Anthocyans)

Food plants that are rich in anthocyanins have a purple, red, blue, or black colour. Also, some of the colors of autumn leaves are due to the presence of anthocyanins [24,25]. Anthocyanins play an antioxidant role against reactive oxygen species (ROS) caused by abiotic stresses, such as overexposure to UV-light [26] and extreme temperatures [27,28]. 

The aromatic rings in the molecular structure of anthocyanins can offer electron density to the empty π* orbital of singlet oxygen. Also the positively charged oxygen atom can accept electron density from the occupied π* orbital of ^1^Δ_g_ (singlet oxygen), the HOMO.

**Case b: Degradation kinetics of anthocyanins [29]**

The authors report on a study about the determination of the degradation kinetic parameters for blackberry anthocyanins in both juice and concentrate during heating and storage at various temperatures [29].

The activation energy value for the degradation of blackberry anthocyanins during heating was found to be 58.95 kJmol^−1^ for the (blackberry) juice. During storage anthocyanin in the blackberry juice concentrate degraded more rapidly than that in blackberry juice with the activation energies being 65.06 kJmol^−1^ and 75.5 kJmol^−1^, respectively. Factors influencing the anthocyanin stability include pH, oxygen, enzymes, light, ascorbic acid, sulfur dioxide or sulphite salts, sugars, metal ions, and copigments [30]. Heat treatment is one of the most important factors that affect the stability of anthocyanins. 

Our calculated values of E_act_, ΔH^≠^, ΔS^≠^ are presented in Table 1. 

ΔG^≠^ = 109.33 kJmol^−1^ at 310 K. We suggest reaction of anthocyanins with oxygen and occupancy of singlet oxygen’s empty π* orbital by anthocyanins’ regions of high electron density. That explains the negative ΔS^≠^ and the numerical value (92 + 17) kJmol^−1^ of ΔG^≠^.

**Case c: Degradation kinetics of anthocyanins [31]**

Anthocyanin stability was tested for seven food products: blood orange juice, two blackberry juices with high and low content of suspended insoluble solids (SIS), and four roselle extracts. The highest content of anthocyanins was found in the blackberry juice. Anthocyanins in blood orange juice degraded with the highest rate constant followed by the blackberry juices and the roselle extracts. Activation energies were found to be 66 kJmol^−1^ for blood orange and 37 kJmol^−1^ for blackberry. For roselle extracts, the activation energies were 47–61 kJmol^−1^. Slight protection for the anthocyanins was provided by a high SIS content for the blackberry juices. Besides their health effects [32], beneficial effects for human diseases have also been referred [33]. The reduction of risks of coronary heart disease, cancer and stroke have been reported [34]. The sensitivity of anthocyanins in the various food products may be depended on the chemical composition, the dissolved oxygen concentration and the chemical structure of anthocyanin.

Our calculations gave: ΔG^≠^ 106.75 (= 92 + 14.75) kJmol^−1^ at 310 K and 108.47 (= 92 + 16.47) kJmol^−1^ at 310 K (case c, Table 1). Reaction of anthocyanin with oxygen takes place and occupancy of singlet oxygen’s empty π* orbital by regions of high electron density of anthocyanins. That is why ΔS^≠^ is negative and ΔG^≠^ is the sum of 92 kJmol^−1^ + the diffusion activation energy.

#### 4.1.3. Carotenoids or Tetraterpenoids

They are categorized as xanthophylls, which contain oxygen and carotenes that are purely hydrocarbons, not containing oxygen. Carotenoids that contain unsubstituted beta-ionone rings and other carotenoids, such as beta-carotene, alpha-carotene, beta-cryptoxanthin, and gamma-carotene, can act as antioxidants. 

Carotenoids contain polyene chain, long conjugated double bonds, which carry out antioxidant activities by quenching singlet oxygen and scavenging radicals to terminate chain reactions in the cell membrane.

So, the biological benefits of carotenoids may be due to their antioxidant properties attributed to their physical and chemical interactions with cell membranes. Their highly unsaturated chemical structure makes them very susceptible to thermal degradation and oxidation.

**Tomato carotenoids:** Tomato carotenoids act as antioxidants to help protect cells and include carotenes and xanthophylls. The provitamin A carotenes are converted to vitamin A inside the body and are important in the eyes (visual cycle). Other carotenoids in tomatoes can also accumulate in the eyes and help to filter damaging rays of light. 

**Xanthophylls**: The main xanthophylls in tomatoes are lutein and zeaxanthin and are yellow pigments. They contain oxygen atoms besides carbon and hydrogen atoms (see above carotenes). Vitamin A, lutein, and zeaxanthin are very important to the health of the eyes. 

**Lutein:** Lutein is a xanthophyll and one of the 600 known naturally occurring carotenoids.

**Zeaxanthin:** Zeaxanthin is one of the two primary xanthophyll carotenoids that are contained within the retina of the eye. Within the central macula zeaxanthin is the dominant component, whereas in the peripheral retina, lutein predominates. 

**Beta-carotene:** β-carotene being highly conjugated is a colored red-orange pigment abundant in plants and fruits. As a hydrocarbon lacking functional groups, it is very lipophilic. Among the carotenes, β-carotene is distinguished by having beta-rings at both ends of the molecule. β-carotene is the most common form of carotene in plants. In nature, β-carotene is a precursor (inactive form) to vitamin A via the action of beta-carotene 15, 15′-monooxygenase [35]. 

Pairs of electrons or electron density in the molecular structure of beta-carotene that can be accommodated to singlet oxygen’s empty π* orbital: the conjugated bonds of the polyene chain are regions of high electron density, and thus they can react with singlet oxygen’s empty π* orbital. They can also trap radicals in the cell membrane. However, as stated above, carotenoids quench singlet oxygen mainly via energy transfer.

**Lycopene:** Lycopene is a phytochemical, a bright red carotene, insoluble in water, which is found in tomatoes and other red fruits and vegetables. Parsley and asparagus contain also lycopene [36] though they are not red as the chlorophyll present in these foods masks the presence of the lycopene. Although chemically lycopene is a carotene, it has no the vitamin A activity [37]. Lycopene may help to prevent breast and prostate cancer. 

The eleven conjugated double bonds are responsible for lycopene’s deep red color and its antioxidant activity in vitro. 

Pairs of electrons or electron density regions in the molecular structure of lycopene that can be accommodated to singlet oxygen’s empty π* orbital: lycopene being a polyunsaturated hydrocarbon bears regions of high electron density, the conjugated double bonds, and thus they can be accommodated in the empty π* orbital. The polyene chain in lycopene can also trap radicals in the cell membrane.

**Case d: Antioxidant activity and carotenoids content** [38]

Carrot has the highest carotene content among foods [38]. The authors studied the influence of air drying temperature on antioxidant activity and carotenoids content to establish the degradation kinetics. The activation energy value that was found and reported is 23.7 kJmol^−1^. The authors concluded that the drying process significantly decreased the carrot antioxidant activity. Our calculations based on the authors’ results are presented in Table 1. ΔG^≠^ = 114.31 kJmol^−1^ at 310 K. Again, singlet oxygen’s empty π* orbital reacts with carotenoids’ regions of high electron density.

**Case e: Degradation of carotenoid antioxidants** [39]

Bioavailability of lycopene is believed to be increased by processing, such as cooking or chopping, because of the breaking down the cell walls, increasing the accessibility of carotenoids [39]. Processing also converts some of the trans-isomers of lycopene to the more bioavailable cis-isomers. Heat and light induces lycopene oxidation and isomerization. The all-trans form isomerises to the cis-form. The aim of the authors’ study was to determine the effect of temperature and time on the degradation kinetics of lycopene and beta-carotene content in tomato paste produced from Roma (i) and Ajindi-Kerewa (ii) tomato cultivars [39]. The activation energies of lycopene degradation obtained from the Arrhenius plots are 8.65 kJmol^−1^ [(ii) cultivar lycopene] and 4.63 kJmol^−1^ [(i) cultivar lycopene]. While the activation energies of lycopene degradation in (ii) tomatoes are twice that in (i) those of beta-carotene degradation are almost the same in the two cultivars: 4.81 kJmol^−1^ [(ii) cultivar beta-carotene] and 4.14 kJmol^−1^ [(i) cultivar beta-carotene]. 

Our calculations give for [(ii) cultivar lycopene], ΔG^≠^ = 101.11 kJmol^−1^, at 310 K. For [(i) cultivar lycopene ΔG^≠^ = 99.48 kJmol^−1^ at 310 K.

The [(ii) cultivar beta-carotene] gives ΔG^≠^ = 100.44 kJmol^−1^ at 310 K.

The [(i) cultivar beta-carotene] gives ΔG^≠^ = 99.41 kJmol^−1^ at 310 K.

The ΔG^≠^ values are almost similar. We suggest similar mechanisms for the two cultivars, the mechanism that we have already suggested for carotenoids and anthocyanins [see also below Section 4.2 mechanism of the studied antioxidants’ reactions with oxygen].

**Case f: Beta-carotene degradation** [40]

The authors’ results indicate that at high concentrations oxygen diffusion becomes a limiting factor in agreement with our mechanism, according to which the studied reaction is the reaction of the antioxidant (beta-carotene) with oxygen. The highly unsaturated chemical structure of carotenoids makes them very susceptible to thermal degradation and oxidation in agreement with our mechanism (Section 4.2), according to which the empty π* orbital of ^1^Δg accommodates electron density from the highly unsaturated chemical structure of carotenoids.

First order kinetics is expected if beta-carotene and oxygen are the only reactants and if a large excess of oxygen is present [40]. Presumably, the carotene is uniformly dispersed in the fatty acid layer through which oxygen has to diffuse before the oxidation can occur. This work propose E_act_ = 22.8 Kcal mol^−1^ that is, 95.4 kJmol^−1^. Our calculations gave E_act_ = 95.73 kJmol^−1^ and ΔG^≠^ = 107.37 kJmol^−1^ at 310 K. Again we suggest the mechanism of Section 4.2. 

**Curcumin:** Curcumin is a chemical produced by some plants. 

Curcumin contains a molecular structure in the presence of hydroxyl and keto moieties on the enol and keto form of the molecule which are responsible for the high antioxidant properties. The unsaturated bonds of the chain trap radicals in the cell membrane, while the terminal rings of curcumin could scavenge radicals at the outer and inner parts of the cell membrane. 

**Case g: Stability of curcumin during storage** [41]

Limited information is available concerning curcumin stability in microencapsulated turmeric oleoresin. There exists a need to investigate the storage stability of this curcumin in inclusion complex that contributes to unique yellow colour of turmeric oleoresin. Activation energy in the presence of light was 23.88 kJmol^−1^ and in the dark was 13.65 kJmol^−1^. Relatively low activation energy obtained in the authors’ study for both with or without light indicates that degradation will continue at a measurable rate at low temperatures, for instance, during storage, leading to a limited shelf life.

Our calculations give: E_act_ = 24.213 kJmol^−1^ and ΔG^≠^ = 94.912 kJmol^−1^ at 310 K for the reactions taking place in the presence of light.

Our calculations give: E_act_ = 11.803 kJmol^−1^, ΔG^≠^ = 99.32 kJmol^−1^ at 310 K for the reactions taking place without the presence of light. We suggest the mechanism of Section 4.2.

According to our results, the degradation in the presence of light (ΔG^≠^ = 94.912 kJmol^−1^ at 310 K) is faster than without the presence of light (ΔG^≠^ = 99.32 kJmol^−1^ at 310 K).

According to the authors’ results, in the dark the degradation is faster (23.88 kJmol^−1^ in the presence of light and in the dark 13.65 kJmol^−1^). This finding cannot be correct and it justifies our suggestion of using ΔG^≠^ instead of E_act_. 

#### 4.1.4. Polyphenols also Polyhydroxyphenols

Polyphenols are molecules with large conjugated systems of π electron configurations bearing aromatic rings. They are reactive towards oxidation possessing a significant binding affinity for proteins, leading to the formation of soluble and insoluble protein-polyphenol complexes [42]. 

Polyphenols, which often have antioxidant properties in vitro, are not necessarily antioxidants in vivo, due to extensive metabolism [43]. The catechol group, which acts as an electron acceptor, is responsible for the antioxidant activity [44] in many polyphenols. 

There are many different structures of phenolic compounds and this makes it difficult to come to broad conclusions about their specific health effects. 

Although in vitro experiments show that polyphenols are good antioxidants, the antioxidant actions in vivo are shown to be probably minor or absent [45] [see in Section 4.1 our comments for case h]. By still undefined mechanisms, flavonoids and other polyphenols may reduce the risk of cancer and cardiovascular disease [46]. Polyphenols in vivo are poorly (less than 5%) conserved with most of the absorbed existing as chemically modified metabolites [47]. The increase in antioxidant capacity of blood observed after the consumption of foods rich in polyphenols has been proposed not to be caused by the polyphenols themselves, but most probably by increased levels of uric acid as derived from the metabolism of flavonoids [47,48]. Generally, foods contain complex mixtures of polyphenols [49]. Some polyphenols interfere with the absorption of essential nutrients, especially iron and other metal ions, and so they are considered antinutrients. They also bind to digestive enzymes and other proteins, particularly in ruminants [50] behaving as antinutrients.

Pairs of electrons in the molecular structure of polyphenols that can be accommodated to singlet oxygen’s empty π* orbital are the oxygen lone pairs the phenyl rings’ electron densities and the unsaturated double bonds’ electron densities wherever they exist.

**Case h: Thermal degradation of polyphenols** [51]

In this study, the authors examined the thermal degradation of the semi-solid residue (grape marc) with high content of antioxidants and its filtered extract [51]. 

Degradation kinetics was studied in terms of total phenol content, antioxidant activity, and anthocyanin content. The activation energy for the grape marc is slightly higher than that for the filtered extract. Due to their diverse structure, different polyphenols are found in the distinct parts of the grape skin and seed. Some of them are free and can be found in vacuoles, while others are associated to cell wall compounds or to polysaccharide structures in the skin cells [51]. 

Thermal treatments on enhanced polyphenol extraction degrade active compounds. The E_act_ for grape marc was found to be 55.980 kJmol^−1^ and for filtered extract 53.196 kJmol^−1^. 

Our calculations give for grape marc ΔG^≠^ = 94.86 kJmol^−1^ at 310 K and for filtered extract ΔG^≠^ = 94.46 kJmol^−1^ at 310 K. These values suggest that the mechanism that we propose is valid for the cases of polyphenols as well. However, in vivo the situation may be different due to the extensive metabolism [43].

**Sulforafane (SF):** Sulforafane is an organosulfur compound. It is found in cruciferous vegetables. 

SF is of interest since it primarily modulates the activities of phase II enzymes that convert carcinogens to inactivate metabolites, thereby preventing them from interacting with DNA [52]. SF can also inhibit histone deacetylase activity in human colorectal and prostate cancer cells, leading to cancer prevention [53,54].

Regions of high electron density in the molecular structure of SF that can be accommodated to singlet oxygen’s empty π* orbital are the unsaturated bonds. The atoms S, N, O bear lone pairs of electrons, which can also be accommodated to singlet oxygen’s empty π* orbital. 

**Case i: Degradation kinetics of sulforaphane** [55]

It has been proved that SF lowers the risks of various cancers, such as breast cancer, lung cancer, colorectal cancer, prostate cancer and/or bladder cancer [55]. SF is not a stable compound, and its stability is affected by pH, temperature, heating time, oxygen, and possibly by some food components [55]. 

This work [55] deals with thermal degradation kinetics of SF in broccoli extract at selected temperatures and pH values [55]. The authors’ results indicated that SF is unstable at high temperatures and is more heat-stable in acidic food products. The ΔG^≠^ values, as calculated by us, based on the results of work [55], are almost the same for the various values of pH, SF being though more stable in acidic pHs (higher values of ΔGs^≠^ implying lower values of rate constants k and thus lower degradation rates meaning more stable SF).

Our calculations based on the authors’ results give (Table 1) for pH = 2.2, ΔG^≠^ = 114.95 kJmol^−1^ at 310 K. For pH = 3.0, ΔG^≠^ = 115.08 kJmol^−1^ at 310 K, for pH = 4.0 ΔG^≠^ = 112.32 kJmol^−1^ at 310 K, for pH = 5.0 ΔG^≠^ = 110.2 kJmol^−1^ at 310 K and for pH = 6.0 ΔG^≠^ = 108.9 kJmol^−1^ at 310 K.

The above values suggest that the mechanism that we propose (Section 4.2) is valid for the case of SF as well. 

**Diallyl disulphide:** Diallyl disulfide is derived from garlic and a few other plants of the genus Allium [56]. Diallyl disulfide, diallyl trisulfide and diallyl tetrasulfide are principal components of the distilled oil of garlic which is insoluble in water yellow liquid. Upon crushing garlic and other plants of the Alliaceae family, allicin is released, the decomposition of which produces diallyl disulfide. Diallyl disulfide is responsible for the many of the health benefits of garlic.

Diallyl disulfide increases the production of the enzyme glutathione S-transferase which acts as a detoxicator of the cells. Garlic protects nerve cells from oxidative stress in vitro [57,58,59,60,61,62,63,64].

By causing detoxification in the liver, diallyl disulfide can offer protection of liver during the chemotherapy [65,66]. 

The organosulfur compounds that are released from Alliaceae plant cells possess antimicrobial, insecticidal and larvicidal properties [67]. The inhibition of the growth of molds and bacteria by garlic oil is due to diallyl disulfide. It also acts against helicobacter pylori allicin acting more efficiently [68,69]. Due to its antimicrobial effects diallyl disulfide together with tobramycin is used in preparations, which are used for the (selective) decontamination of the organs before surgical operations. Such preparations prevent endotoxemia in heart valve operations [70]. 

Diallyl disulfide is responsible for garlic’s action in preventing the colorectal cancer [71]. Diallyl disulfide affects cancer cells more than normal cells [72]. Garlic may protect from the development of cardiovascular diseases. Oxidative stress may be a reason for some of these diseases, such as atherosclerosis or coronary heart disease. Diallyl disulfide reduces oxidative stress by participating in the detoxification of the cell along with some other mechanisms [73]. Diallyl disulfide can also lead to a short-term lowering of blood pressure [74] by activating the TRPA1 ion channel.

In a review article titled “Herbal medicine for the treatment of cardiovascular disease (clinical consideration)” [75], the authors refer to the important qualities of garlic. Also, the effect of a garlic oil preparation on serum lipoproteins and cholesterol metabolism was reported [76]. Promise for improving some cardiovascular risk factors by garlic’s properties is referred in [77]. There are also reports with title “Garlic prevents plaque” [78] and “garlic prevents blood clots” [79]. 

Pairs of electrons in the molecular structure of diallyl disulphide that can be accommodated to singlet oxygen’s empty π* orbital are the sulfur lone pairs and the double bonds’ electron density. 

**Thiosulfinate:** Thiosulfinate is a functional group consisting of the linkage R-S(O)-S-R (R are organic substituents).

Pairs of electrons in the molecular structure of thiosulfinate that can be accommodated to singlet oxygen’s empty π* orbital are O and S lone pairs of electrons.

**Case j: Antioxidant activity and degradation of thiosulfinates of garlic** [80]

In the work [80], the rate constants for loss of thiosulfinate and for loss of antioxidant activity were found to increase with blanching temperature. The activation energies were 7.67 kJmol^−1^ and 89.75 kJmol^−1^, respectively. The antioxidant activity was correlated with thiosulfinates and the antioxidant activity, along with the thiosulfinate contents were decreased with increasing blanching time. Banerjee et al. [81] support that thiosulfinates not only have antioxidant activity, but also can stimulate the synthesis of glutathione, which is an important antioxidant. Heat treatment of garlic affects the antioxidant activity of thiosulfinate because of the inactivation of alliinase, which is responsible for the formation of these compounds through interaction with alliin [82]. The antioxidant activity may be associated with the presence of phenolic compounds such as flavonoids and mericitin [83].

Antioxidant Activity: a loss of antioxidant activity was observed over time during blanching. This results from the damage to the plant tissue by heating, which leads to the exposure of antioxidant compounds [84]. Yin and Cheng [85] reported that the activity of organosulfur compounds is lowered by high temperatures due to inactivation by heat of alliinase, the enzyme that is responsible for the formation of thiosulfinates [86]. This confirms the high loss of these compounds in the presented study. It was suggested that the antioxidant activity was due to thiosulfinates and to other compounds such as the phenolics, which were not evaluated in the study.

Our calculations gave for antioxidant activity ΔG^≠^ = 101.21 kJmol^−1^ at 310 K, and for loss of thiosulfinates ΔG^≠^ = 91.27 kJmol^−1^ at 310 K. The value of ΔG^≠^ for loss of thiosulfinates could suggest that there is no need for diffusion for this process to take place, something that is expected. It could also mean that singlet oxygen is not involved here.

The calculated values suggest that the proposed mechanism (Section 4.2) is valid for the case of the antioxidant activity and thiosulfinate degradation of garlic as well. 

**Vitamin C:** Vitamin C (Ascorbic acid) is a water soluble monosaccharide. During primate evolution, one of the enzymes needed to synthesize ascorbic acid (enzyme gulonolactone oxidase) has been lost by mutation. Humans and apes must obtain it from the diet since it is a vitamin [87]. Most other animals being able to produce it in their bodies do not require it in their diets [88]. It is essential for collagen, carnitine, and neurotransmitters biosynthesis [89]. By reaction with glutathione, it remains in its reduced form. Ascorbic acid, being a redox catalyst, can reduce and thus neutralise, reactive oxygen species (ROS) such as hydrogen peroxide [90] (antioxidant action). 

Pairs of electrons in the molecular structure of vitamin C that can be accommodated to singlet oxygen’s empty π* orbital are the lone pairs of oxygen atoms. Regions of high electron density are also the double bonds.

**Case k: Vitamin C degradation** [91]

Various vitamin C tablet samples were examined e.g., standard ascorbic acid solution (Merck), vitamin C 200 mg tablets, Ascovit 100 mg tablets with orange taste and vitamin C nose drops 10%. The variations of vitamin C concentration were studied as a function of light exposition at various temperatures. The values of E_act_ that were found are: standard ascorbic acid solution.

(Merck) 8.73 kcalmol^−1^ (36.53 kJmol^−1^), 200 mg tablet 6.65 kcalmol^−1^ (27.82 kJmol^−1^), Ascovit 100 mg tablet 2.62 kcalmol^−1^ (10.96 kJmol^−1^), Vitamin C nose drops 8.39 kcalmol^−1^ (35.10 kJmol^−1^). 

The calculated by us values of ΔG^≠^ are: standard ascorbic acid solution (Merck) ΔG^≠^ = 97.79 kJmol^−1^ at 310 K, 200 mg tablet ΔG^≠^ = 93.6 kJmol^−1^ at 310 K, Ascovit 100 mg tablet ΔG^≠^ = 94.26 kJmol^−1^ at 310 K, Vitamin C nose drops ΔG^≠^ = 95.73 kJmol^−1^ at 310 K. 

Thus, the ΔG^≠^ values for the various samples were very similar and very close to the value of 92 kJmol^−1^, that is, close to the energy that is needed for the excitation of ground state oxygen to the first excited state, the singlet oxygen state.

**Case l: Temperature and high pressure effect on lycopene and vitamin C** [92]

The aim of the study was to determine the kinetics of vitamin C and lycopene degradation of watermelon juice under various temperatures and high pressures [92]. According to the authors in the conditions of processing (thermal and high pressure), lycopene is found to be more stable than vitamin C. Thermal treatment resulted in faster degradation rates of the two components compared to the high pressure treatment. It has been found that there is a relation between lycopene consumption with diet and lower incidence in prostate and oral cancers. It may also lower the risks of cardiovascular disease [92]. Vitamin C is unstable towards oxygen, heat, light, and metal catalysts. Lycopene changes during processing and storage. It undergoes oxidation and isomerization from all-trans to mono-cis or poly-cis forms [92]. In fruits and vegetables the all-trans isomer is the most predominant and is also the most thermodynamically stable form [92]. The thermal degradation of vitamin C is faster than that of lycopene. So, the E_act_ value for the thermal degradation of vitamin C of watermelon juice was found to be 18.37 kcalmol^−1^ (76.86 kJmol^−1^) whereas the corresponding value of lycopene of watermelon juice was found to be 23.35 kcal mol^−1^ (97.7 kJmol^−1^).

Our calculations give for E_act_ of vitamin C degradation 76.92 kJmol^−1^ and for ΔG^≠^ = 105.528 kJmol^−1^ at 310 K. The E_act_ of lycopene degradation is found to be 97.21 kJmol^−1^, and the ΔG^≠^= 116.46 kJmol^−1^ at 310 K.

The above presented values suggest that the proposed mechanism (Section 4.2) is valid also for the case of lycopene and vitamin C.

**Case m: Oxidation of vitamin C** [93]

Vitamin C is important in the functioning of the brain. Fruits and vegetables like oranges, berries, potatoes, greens, and tomatoes are the main sources of vitamin C for humans. The oxidation of vitamin C occurs very quickly in a basic environment at high temperatures. The amount of vitamin C is significantly reduced if it is stored at room temperature [93]. The results of the authors’ research indicate that between 40 and 80 °C the kinetics of the oxidation of ascorbic acid, vitamin C, is a first order reaction and the activation energy for this reaction was found to be 20.73 kJmol^−1^. Our calculations, according to the authors’ results, gave the following values: E_act_ = 41.65 kJmol^−1^ and ΔG^≠^ = 108.47 kJmol^−1^ at 310 K.

Again the proposed mechanism (Section 4.2) is valid also for the case of vitamin C oxidation.

**Cysteine:** Cysteine is a semi-essential proteinogenic amino acid. The thiol (–SH) side chain in cysteine often participates in enzymatic reactions. It is oxidized to give the disulfide (–S–S–) derivative cystine, which plays an important structural role in many proteins. 

Cysteine has antioxidant properties due to the ability of thiols to undergo redox reactions. Cysteine’s antioxidant properties are expressed in the tripeptide glutathione, which occurs in humans as well as other organisms. Glutathione is biosynthesized from its constituent amino acids, cysteine, glycine, and glutamic acid. Cysteine is an important source of sulfide in human metabolism. 

Pairs of electrons in the molecular structure of cysteine that can be accommodated to singlet oxygen’s empty π* orbital are lone pairs on O, N and S atoms. 

#### 4.1.5. Fumaric Acid or Trans-Butenedioic Acid

Fumaric acid activates the Nrf2 antioxidant response pathway, the primary cellular defense against the cytotoxic effects of oxidative stress [94]. 

Pairs of electrons in the molecular structure of fumaric acid that can be accommodated to singlet oxygen’s empty π* orbital are the O lone pairs and the conjugated double bonds’ electron density.

**Case n: Effect of antioxidants [vitamin C, cysteine, fumaric acid] on the stability of an HTK (histidine-tryptophane-α-ketoglutaric acid) solution** [95]

The study aimed at determining the stability of an HTK (histidine-tryptophane-α-ketoglutaric acid, solutions in transplantology) solution using the changes of the histidine content i.e., the stability of the amino acid as affected by various antioxidants (vitamin C, cysteine, fumaric acid). When solutions containing histidine are stored, the process of the compound’s oxidation takes place, according to the authors, who support that the reaction consists mainly of the addition of the free radical to the aromatic ring. The authors state, that this is a relatively quick reaction as the resulting free radical adducts are stabilized by the resonance action of the aromatic ring [95]. The study shows that vitamin C is the most effective antioxidant.

E_act_ reported by the authors: HTK 23.945 cal mol^−1^ (100.186 Jmol^−1^). Our calculations gave for ΔG^≠^ = 113.92 kJmol^−1^ at 310 K. So, we believe that the units of the results for all cases in [95] are not calmol^−1^ but kcalmol^−1^.

HTK + cysteine, E_act_ = 21.980 cal mol^−1^ (91.96 Jmol^−1^). Our calculations gave for ΔG^≠^ = 114.96 kJmol^−1^ at 310 K. 

HTK + vitamin C, E_act_ = 23.453cal mol^−1^ (98.127 Jmol^−1^). Our calculations gave for ΔG^≠^ = 115.61 kJmol^−1^ at 310 K. 

HTK + fumaric acid, E_act_ = 21.440 cal mol^−1^ (89.70 Jmol^−1^). Our calculations gave for ΔG^≠^ = 114.87 kJmol^−1^ at 310 K. 

Vitamin C is proved to be a more effective antioxidant since ΔG^≠^ is the highest, and thus the rate constant (of oxidation) is the lowest, and so the degradation (oxidation) is the slowest.

The proposed mechanism (Section 4.2) is, according to the above results, valid also for the cases of the antioxidants vitamin C, cysteine and fumaric acid. 

**Walnut oil:** Walnut oil is oil extracted from walnuts Juglans regia (Persian walnuts). Walnuts are rich in phytonutrients being excellent sources of phosphorous, selenium, magnesium, iron, zinc, and calcium. Walnuts and/or walnut oil provide big levels of Vitamins B_1_, B_2_, and B_3_, along with Vitamin E and niacin. 

Niacin, known also as nicotinic acid, together with nicotinamide, it makes up the group known as vitamin B_3_ complex. 

The oil provides polyunsaturated fatty acids, monounsaturated fatty acids, and saturated fatty acids. Unlike most nuts that are high in monounsaturated fatty acids, walnut oil is composed largely of polyunsaturated fatty acids, particularly alpha-linolenic acid and linoleic acid, and it does contain oleic acid [96]. It does not contain cholesterol.

Walnut oil is edible, and, like all nut seed and vegetable oils will undergo rancidification accelerated by heat, light and oxygen.

Pairs of electrons in walnut oil constituents that can be accommodated to singlet oxygen’s empty π* orbital are the oxygen, nitrogen and sulfur lone pairs and the aromatic rings’ and double bonds’ electron densities. 

**Case o: Kinetic parameters of walnut oil** [97]

Rancimat was one of the tests that were developed for testing the resistance to oxidation of fats. Being easy for use and due to its repeatability has become a popular method that is used up to now [98,99]. Rancimat test allows for estimating the induction period (IP) or the oxidative stability index (OSI), which is the period before oil rapid oxidation. The oxidation reaction rate constants (k) of walnut oil at various temperatures were determined. The presence of double bonds increases the oxidation rate [100]. This is explained by the mechanism that we propose as follows: the double bonds’ electron densities react with the empty π* orbital of singlet oxygen. The activation energy E_act_ of walnut crude oil was determined to be 80.327 kJmol^−1^. The amount of polyunsaturated fatty acids (linoleic, linolenic, and oleic acids) in vegetable oils has decisive role in their oxidative stability so that by increasing the polyunsaturated fatty acids, the oil oxidative stability is reduced [101]. According to our mechanism (Section 4.2), the polyunsaturated acids react with singlet oxygen’s empty π* orbital through the electron densities of the unsaturated bonds. The presence of polyunsaturated fatty acids in vegetable oils affect their activation energies. In general, it has been reported that high levels of polyunsaturated acids (linoleic acid and linolenic) reduce the activation energy, (increase of decomposition rate due to unsaturated bonds that react with the empty π*). The value of enthalpy and entropy of activation of walnuts crude oil was found by the authors to be ΔH^≠^ = 77.27 kJmol^−1^ and ΔS^≠^ = −55.76 JK^−1^mol^−1^, respectively. The E_act_ = 80.32 kJmol^−1^, the ΔG^≠^ = 94.56 kJmol^−1^ at 310 K calculated by us according to the ΔH^≠^ and ΔS^≠^ reported by the authors. 

Our calculations gave: E_act_ = 10.04 kJmol^−1^, ΔH^≠^ = 6.99 kJmol^−1^, ΔS^≠^ = −307.8 JK^−1^mol^−1^, ΔG^≠^ = 102.4 kJmol^−1^ at 310 K.

The differences in the calculated values of the thermodynamic parameters are due to the different units of the rate constants that were expressed by the authors in the unit “per hour”. They should be expressed “per second” since the Planck’s constant is expressed in Joule.sec. The Planck’s constant is involved in the Eyring equation.

#### 4.1.6. Oxygen-Free Reaction of Diallyl Disulfide

**Case p: Kinetics and mechanism of diallyl disulfide thermal decomposition** [102]

The work deals with diallyl disulfide pyrolysis, that is, reaction under oxygen-free conditions. Diallyl disulfide is present in garlic oil and posseses anticarcinogenic properties [103,104].

In this work [102] the experimental system was evacuated, that is, the experiments were designed in such a way that, there was no reaction with oxygen. This means that our mechanism that are valid in the presence of O_2_ cannot be applied in this case where the mechanism must be different and the thermodynamic parameters would have different values. Especially the ΔG^≠^ does not correspond to reaction with singlet oxygen. The ΔG^≠^ value is different from the values for the other cases (a–o) reported in this paper which are: (a) similar; (b) they have values equal to 92 kJmol^−1^ + E_act_ for diffusion. The ΔG^≠^ for this case p is 135.81 kJmol^−1^ at 310 K, completely different from the values for all of the cases of the food antioxidants that were studied that range between 91.27 and 116.46 kJmol^−1^ at 310 K.

Analytically our calculations based on the authors’ results give: E_act_ = 123.32 kJmol^−1^, ΔH^≠^ = 119.60 kJmol^−1^, ΔS^≠^ = −52.30 JK^−1^mol^−1^, ΔG^≠^ = 135.81 kJmol^−1^ at 310 K, a completely different from the other cases value.

In addition to the above case p we searched for more cases of reactions of antioxidants under oxygen-free conditions in order to add weight to the conclusion that singlet oxygen is involved in the degradation process. The following cases (a), (b), (c) lead to the conclusion that the ΔG^≠^ becomes higher under oxygen-free conditions:

(a) The kinetics of quercetin (a plant polyphenol from the flavonoid group) oxidation in the absence of air [105]. The authors state that: “The results, … show clearly that the absence, …. or at least substantial reduction, of oxygen content markedly slows down quercetin oxidation.” This means that quercetin oxidation becomes slower, that is, the ΔG^≠^ value becomes higher.
(b) “Circulating air increased the rate of lycopene degradation” [106]. This suggests that the absence of oxygen decreased the degradation, which means that increased the ΔG^≠^ value.
(c) “Oxygen-free conditions reduce lycopene losses significantly” [107]. This means that without the presence of oxygen lycopene is degraded more slowly meaning that the ΔG^≠^ value is higher.

**Conclusion**: Except for the case (a), for which we found positive ΔS^≠^ and we suggest a composite mechanism, all other values of ΔS^≠^ are negative which agrees with the mechanism that we propose, according to which the lone pairs of electrons or the electron densities of the antioxidants are accommodated at the singlet oxygen’s empty π* orbital. This procedure happening at the rate determining step leads to a higher organization in the transition state than in the reactants, that is, leads to less degrees of freedom, since the two species become one. 

Other important Antioxidants for which we had no available data for our calculations are:

**Astaxanthin** (C_40_H_52_O_4_) is an antioxidant that crosses the blood brain barrier. It has been reported by Naguib et al. [108] that astaxanthin has higher antioxidant activity compared to various carotenoids such as α-carotene, lutein, lycopene and β-carotene. Actually antioxidant activity of astaxanthin was 10 times more than zeaxanthin, lutein, canthaxanthin, β-carotene and 100 times higher than α-tocopherol [109]. 

Astaxanthin contains a molecular structure in the presence of hydroxyl and keto moieties on each ionone ring, which are responsible for the high antioxidant properties [108,110]. The polyene chain in astaxanthin traps radicals in the cell membrane, while the terminal ring of astaxanthin could scavenge radicals at the outer and inner parts of the cell membrane. So, astaxanthin’s molecular structure enables it to stay both in and outside the cell membrane. Vitamin C and β-carotene can be positioned inside the lipid bilayer so astaxanthin gives better protection than the above two antioxidants. 

Astaxanthin can be found in high concentrations within the retina. It has been reported that astaxanthin, along with the similar fat-soluble carotenoids lutein and zeaxanthin, may help in absorbing some of the blue light and near-UV rays that the retinas are exposed to daily. There are studies suggesting that aside from cataracts and macular degeneration astaxanthin benefits include cardiovascular health, brain aging, cancer, diabetes, as well as other diseases and conditions [111].

**Coenzyme Q-10** is produced naturally in our body. It crosses the blood brain barrier. It is used for cell growth and to protect cells from damage that could lead to cancer.

The oxidized molecule that has three isoprenoid units is called Q3.

CoQ10 contains a molecular structure in the presence of keto moieties on ring, which are responsible for the high antioxidant properties. The polyene chain in CoQ10 traps radicals in the cell membrane, while the terminal ring of CoQ10 could scavenge radicals at the outer and inner parts of cell membrane. 

**Glutathione (GSH)** is an important antioxidant in some bacteria and archaea, in fungi, plants, and animals. Glutathione prevents damage to important cellular components that are caused by reactive oxygen species, such as free radicals, heavy metals, peroxides, and lipid peroxides [112]. Glutathione, serving as an electron donor reduces disulfide bonds of proteins to cysteines. During this process, glutathione is oxidized to glutathione disulfide (GSSG/L-(–)-glutathione). Oxidized glutathione can be reduced back by NADPH (an electron donor) and glutathione reductase [113]. A measure of cellular oxidative stress is often used the ratio of reduced to oxidized glutathione within cells [114,115]. 

Glutathione is one of the most important antioxidants in cells due to its high concentration and its central role in maintaining the cell’s redox state [116]. 

Pairs of electrons in the molecular structure of glutathione that can be accommodated to singlet oxygen’s empty π* orbital are the lone pairs of O, N, S, the double bonds’ electron density. Also, the positively charged nitrogen atom can accept electron density from the occupied π* orbital of ^1^Δ_g_ (singlet oxygen), the HOMO.

Lipoic acid (LA), also known as α-lipoic acid (ALA) and thioctic acid is an organosulfur compound derived from octanoic acid. LA contains two sulfur atoms connected by a disulfide bond and is thus considered to be oxidized.

Lipoic acid is an antioxidant. Although the body can synthesize LA, it can also be absorbed from the diet. Maximum blood levels of LA are achieved 30–60 min after dietary supplementation, and it is thought to be largely metabolized in the liver [117]. In Germany, LA is approved as a drug for the treatment of diabetic neuropathy since 1966 [118]. 

Pairs of electrons in the molecular structure of lipoic acid that can be accommodated to singlet oxygen’s empty π* orbital are the lone pairs of O and S, and the double bond’s electron density.

**Melatonin, N-acetyl-5-methoxy, tryptamine,** is a hormone that is produced by the pineal gland in animals and regulates sleep and wakefulness [119]. In animals, melatonin is involved in the synchronization of the circadian rhythms including sleep-wake timing, seasonal reproduction, blood pressure regulation, and many others [120]. It also functions as synchronizer of the biological clock. In animals, many of its biological effects are due to its role as an antioxidant [121]. It plays a particular role in the protection of nuclear and mitochondrial DNA [122]. 

Melatonin is a powerful wide-spectrum antioxidant [123,124] that easily cross the blood-brain-barrier [121,125,126] and cell membranes. This antioxidant is a direct scavenger of radical oxygen and nitrogen species, including OH•, O_2_^−^•, and NO• [127,128]. Melatonin, once oxidized, upon reacting with free radicals cannot be reduced to its former state because it forms several stable end-products. For this reason it has been referred to as a terminal (or suicidal) antioxidant [129]. It has been proven that melatonin is twice as active as vitamin E, and is believed to be the most effective lipophilic antioxidant [130]. A very important characteristic of melatonin is that its metabolites are also scavengers in what is referred to as the cascade reaction [123]. When melatonin is compared to synthetic, mitochondrial-targeted antioxidants (MitoQ and MitoE), it is proved to be a comparable protector against mitochondrial oxidative stress [131]. 

Pairs of electrons in the molecular structure of melatonin that can be accommodated to singlet oxygen’s empty π* orbital are the lone pairs of O and N, and the electron densities of the rings and of the double C=O bond.

**Vitamin E** is the name for eight related fat-soluble vitamins tocopherols and tocotrienols, with antioxidant properties [132,133]. The body preferentially absorbs and metabolises the form α-tocopherol [134] which has the highest bioavailability. Its action is protecting membranes from oxidation by reacting with lipid radicals that are produced in the lipid peroxidation chain reaction [132,135]. During lipid peroxidation, singlet oxygen is produced [1]. Vitamin E removes the free radicals and stops the propagation reaction. The result of this reaction is oxidized by singlet oxygen α-tocopheroxyl radicals that can be recycled back to the active reduced form through reduction by other antioxidants, such as ascorbate, retinol or ubiquinol [136]. It has been suggested that the most important function of α-tocopherol is as a signaling molecule having no significant role in antioxidant metabolism [137,138]. 

**Tocotrienols** are a rarer form of Vitamin E. It crosses the blood brain barrier. Tocotrienols may be important in protecting neurons from damage [139]. 

Pairs of electrons in the molecular structure of vitamin E that can be accommodated to singlet oxygen’s empty π* orbital are the oxygen lone pairs and the phenyl ring’s electron density. According to its structure, vitamin E may not be a significant antioxidant since there are not many regions of high electron density to capture singlet oxygen’s empty π* orbital (see below “mechanism of action” of the studied antioxidants).

**Vitamin K_2_** has recently been revealed as an important nutrient in protecting against heart disease [140]. It achieves this by guiding the body to add the calcium in the bones and teeth and not in the arteries and soft tissue. The structures of both vitamins K_1_ and K_2_ show why K_2_ can act as an antioxidant, whereas K_1_ not as a good one. 

Regions of high electron density are the double bonded oxygens that bear pairs of electrons that can be accommodated in the singlet oxygen’s empty π* orbital. This also applies for the chain double bonds that exist in K_2_ but not in K_1_. The molecular structure bears the presence of keto moieties on the ring, which are responsible for high antioxidant properties. The polyene chain in vitamin K_2_ traps radicals in the cell membrane, while the terminal ring of vitamin K_2_ could scavenge radicals at the outer and inner parts of cell membrane.

### 4.2. Mechanism of the Studied Antioxidants’ Reactions with Oxygen

The results of our calculations that are presented in Table 1 suggest that for all of the cases (a–o), the free energy of activation could be the sum of the 92 kJmol^−1^ and the E_act_ of diffusion of the reactants. As in the already studied reactions of proteins of proteinaceous diseases the free energy of activation reveals that the rds is the production of singlet oxygen which then reacts with proteins, DNA, biomolecules etc. In the present work again the rds is found to be the formation of singlet oxygen which then reacts with the antioxidants. Examining the structures of the various antioxidants, we can conclude that this might be the case: the various antioxidants, like the proteins of proteinaceous diseases, bear regions of high electron density (basic properties), and thus they can react with the empty π* orbital of singlet oxygen (strong acidic properties). So, there is a possibility of the two following reactions taking place in the two different systems: (a) Proteins, DNA, biomolecules + singlet oxygen’s empty π* orbital, and (b) antioxidants + singlet oxygen’s empty π* orbital. For these two cases the rates of the reactions are:

For both cases: Vrds = k [O_2_] = cT exp − (92/RT). [O_2_], Rds reaction: ^3^Σ_g_^−^ O_2_ → ^1^Δg O_2_

(a) For the reactions of biomolecules with the produced at the rds singlet oxygen:

V_(biomolecules+singlet oxygen’s empty π* orbital)_ = k_(b+so)_ [^1^O_2_] [biomolecules] = c. T. exp (−ΔG^≠^_(b+so)_/RT) [^1^O_2_] [biomolecules], where ^1^O_2_ is the singlet oxygen, (b + so): (biomolecules + singlet oxygen).

(b) For the reactions of antioxidants with the produced at the rds singlet oxygen:

V_(antioxidants+singlet oxygen’s empty π* orbital)_ = k_(a+so)_ [^1^O_2_] [antioxidants]= c. T. exp (−ΔG^≠^_(a+so)_/RT) [^1^O_2_] [antioxidants], where ^1^O_2_ is the singlet oxygen, (a + so): (antioxidants + singlet oxygen).

The values of ΔG^≠^_(b+so)_ and ΔG^≠^_(a+so)_ are almost similar (see Figure 1) the ΔG^≠^_(a+so)_ being slightly smaller which implies slightly higher rate of reaction if the concentrations [biomolecules] and [antioxidants] and k_(b+so)_ and k_(a+so)_ were the same. Assuming that the k_(b+so)_ and k_(a+so)_ are very big and similar since they correspond to reactions of an excited state, ^1^Δ_g_ (with acidic properties) and molecules bearing basic properties then the decisive role that is played by the relation of the concentrations of [biomolecules] and of [antioxidants]. Unfortunately the [biomolecules] is a lot higher than the [antioxidants]. Thus, the rate of reaction of biomolecules is a lot higher than the rate of the reaction of antioxidants. In most of the antioxidants, there are more than one sites of attack by singlet oxygen because they bear many regions that can react, for example, many double bonds, many lone pairs of electrons due to the presence of oxygen(s), nitrogen(s), or sulfur atoms etc. This multiplies the “concentration” of the antioxidants (statistical kinetics). However, the same applies for the “concentration” of the biomolecules (proteins, DNA, etc.): it is multiplied because of the existence of many regions of high electron density due to many lone pairs of electrons in the many oxygen, nitrogen, and sulfur atoms in their molecules. Concluding, the solution is to increase the “concentration” of the antioxidants in order to create:

V_(biomolecules+singlet oxygen’s empty π* orbital)_ < V_(antioxidants+singlet oxygen’s empty π* orbital)_

and thus the antioxidants to win the competition with proteins, DNA, etc., in capturing singlet oxygen’s empty π* orbital.

In Figure 2, the barriers ΔG^≠^ are equal to 92 kJmol^−1^ plus the E_act_ of the reactants’ diffusion. In the case of the biomolecules’ reactions the E_act_ of the diffusion is bigger than the E_act_ of the diffusion of the antioxidants’ reactions due to the nature of the corresponding medium (cell vs. water).

It must be pointed out that our calculated thermodynamic parameters of activation provide only information about the rate determining step (rds), and actually about the value of the free energy of activation. This free energy of activation is equal to 92 kJmol^−1^ + the E_act_ for diffusion. So, we have evidence that singlet oxygen is produced during the rds. We have no evidence of what is next happening. Either a direct reaction of ^1^O_2_ or a reaction by a secondary ROS (Reactive Oxygen Species) is taking place. The observations that are referred to Section 1.2, in paragraph “Singlet oxygen quenching” apply for our findings as well.

Also, our above derived conclusions are in perfect agreement with previous reports on the scavenging activity of certain antioxidants for singlet oxygen [8,9,10,12].

### 4.3. Questions That Need to Be Answered

If the above-suggested mechanism of the role of singlet oxygen, O_2_ (^1^Δ_g_) in the reactions of antioxidants is actually valid, then some questions arise that need to be answered: (1) which are the sources of singlet molecular oxygen in the biological systems? [1]; (2) where does the human body find the energy of 92 kJmol^−1^ for ground state oxygen’s excitation to the first excited state, i.e., to generate singlet oxygen state, ^1^Δ_g_ in a cell? [1]. It is known that the energy of the ATP (phosphoric) bond is 30.66 kJmol^−1^. Can the excitation energy of O_2_ be taken from the use of 3–4 ATP molecules? [1]; (3) is the generation of singlet oxygen increased selectively in neurons, and if so, how does this happen? [1]. The antioxidants must meet neurons if they are going to react with singlet oxygen generated in their environment. Can antioxidants meet neurons? Can they pass the Blood-Brain Barrier (BBB)? 

Which are the sources of singlet molecular oxygen in the biological systems? Potential sources of singlet molecular oxygen in the biological systems have been in detail presented in [1]. Besides other sources, lipid, phospholipid and cholesterol hydroperoxides are sources of singlet molecular oxygen [1].

Where does the human body find the energy of 92 kJmol^−1^ for ground state oxygen’s excitation to the first excited state, i.e., to generate singlet oxygen state, ^1^Δ_g_ in a cell? This energy can be found in a cell according to Peter Mitchell’s “the chemiosmotic hypothesis” [1].

Is the generation of singlet oxygen increased selectively in neurons and if so, how does this happen? This question has also been answered in [1]. The antioxidants must meet neurons if they are going to react with singlet oxygen generated in their environment. Can antioxidants meet neurons? Can they pass the BBB? 

Blood–Brain Barrier (BBB), The BBB is an important filter that protects the brain [141,142] and the cells of the barrier allow, by passive diffusion, the passage of water, lipid-soluble molecules, and some gases, as well as the selective actively transport of molecules (metabolic products), such as glucose (across the barrier with specific proteins) [143] and amino acids that are necessary for neural function. It also prevents the entry of potential lipophilic neurotoxins by way of an active transport mechanism that is mediated by P-glycoprotein. Endothelial cells restrict the diffusion of microscopic objects (e.g., bacteria) and large or hydrophilic molecules into the cerebrospinal fluid (CSF), while they allow for the diffusion of hydrophobic molecules (O_2_, CO_2_, hormones) [144]. It is claimed that astrocytes are necessary to create the blood–brain barrier. A protective layer is played by a thick membrane and astrocytic end feet [145,146]. In addition to the above protection, cerebral endothelial cells have a defense system against oxidative stress, which includes GSH, glutathione peroxidase, glutathione reductase, and catalase when compared to the rest of the brain [147]. In particular, GSH is supported to play a role in maintenance of the BBB integrity [148]. All parts of the healthy brain contain antioxidants like superoxide dismutase (SOD) in order to balance against the produced high concentration of ROS and RNS [149]. Few regions in the brain do not have a BBB, including the circumventricular organs. The neuronal membrane contains polyunsaturated fatty acids especially docosahexaenoic acid. Polyunsaturated fatty acids undergo lipid peroxidation. Cerebral endothelial cells have a high concentration of mitochondria, leading to the generation of oxidative stress [150]. The high concentration of mitochondria is supported to play a role in maintenance of BBB. It has been reported [151] that with aging a decrease in mitochondrial number at cerebral endothelial cells and a subsequent loss of BBB integrity take place. The properties that can cause increase of oxidative stress lead not only to neurodegeneration and breakdown of the BBB via disruption of tight junction proteins, but can also affect blood flow [152]. The brain, particularly the cerebrovascular system, features many sources of producing oxidative stress as well as targets vulnerable to this stress allowing for a cycle of damage.

Does Disruption of the Blood–Brain Barrier Take Place in Alzheimer’s Disease? There is evidence indicating that disruption of the blood–brain barrier in Alzheimer’s disease patients allows blood plasma containing Aβ amyloid to enter the brain where the Aβ adheres preferentially to the surface of astrocytes [153]. These findings have led to some hypotheses about mechanisms in some cases of Alzheimer’s disease. Thus, in some patients, Alzheimer’s disease may be caused (or more likely, aggravated) by a breakdown in the blood–brain barrier [154].

### 4.4. Future Strategies 

Having presented the above (possible) role of singlet oxygen (major component of oxidative stress) in the reactions of antioxidants, we conclude that antioxidant administration or donation of reagents bearing regions of high electron density (bases) may be useful in the prevention and treatment of neurodegenerative diseases and the other disorders that are mentioned above and in [1]. To obtain efficacy in delaying diseases progression, the candidate antioxidant or base must be given as early as possible, before irreversible neuronal loss. The antioxidant or base should also be tailored to the place of generation of ^1^O_2_ (^1^Δ_g_), i.e., to neurons that are rich in lipids and/or metal ions. The chosen antioxidant or the basic properties bearing reagent should also be able to penetrate the blood-brain barrier after systemic administration in order to attain a critical therapeutic level within the CNS [1].

The antioxidants should be ones that could prevent lipid, protein, DNA peroxidation, and thus prevent the production of the ^1^O_2_ (^1^Δ_g_). Suitable bases should be ones that could donate pair of electrons in the empty π* orbital of the produced O_2_ (^1^Δ_g_) in order to prevent the reaction of it (π* orbital) with protein, lipid, DNA, biomolecule in general. 

The bases (reagents bearing regions of high electron density) and the antioxidants should react with the ^1^O_2_ (^1^Δ_g_) faster than lipids, proteins, DNA, etc. Thus,

V_(biomolecules+singlet oxygen’s empty π* orbital)_ < V_(antioxidants+singlet oxygen’s empty π* orbital)_

The bases and the antioxidants must also meet neurons hence they must be able to pass the BBB. In order to achieve what we have found from our calculations, the solution is to increase the concentration of the antioxidants in order to create a higher rate of their reaction with the empty π* orbital of the produced O_2_ (^1^Δ_g_).

Then, the antioxidants will win the competition with proteins, DNA, etc., in capturing singlet oxygen’s empty π* orbital. Thus we must find a way to increase the concentration of the antioxidants in the brain. 

We are trying at the moment to find which antioxidants can have the best overlapping of their orbitals with the singlet oxygen’s empty π* orbital. 

Computational studies have been reported in the literature [155] for the oxidation of guanine by singlet oxygen (^1^Δ_g_) and formation of guanine: lysine cross-links. We have already referred to the molecule of guanine as being high electron density regions of DNA [1].

We are also studying aspirin as a candidate antioxidant since our preliminary calculations showed that the structure of its molecule having regions of high electron densities must have strong antioxidant properties. The valuable role of aspirin in tumor cells has been reported [156].

## 5. Conclusions

The values of ΔG^≠^ suggest that singlet oxygen production (^1^O_2_, ^1^Δ_g_) from ground state oxygen (O_2_, ^3^Σ_g_^−^) demanding 92 kJmol^−1^, may be the crucial/decisive step in the common mechanism of reactions of the various antioxidants that were studied. About 10–30 kJmol^−1^ is the activation energy that is needed for the diffusion of reactants, where this is necessary, depending on the reaction and the medium. In aqueous solutions or in experiments in vitro, the E_act_ of diffusion of the reactants is smaller than in reactions in vivo. We attribute the common mechanism to the same role of oxidative stress and specifically of singlet oxygen to the above mentioned (studied) processes of antioxidants. 

The ability of accepting a pair of electrons in the empty π* orbital gives to the singlet oxygen species, ^1^Δ_g_, strong acidic properties meaning that it can react with antioxidants bearing regions of high electron density, that is, regions of high basicity.

Regions of high electron density in the various antioxidants that were studied are the double bonds, the lone pairs of electrons in O, N, S atoms, the aromatic rings etc., accommodating their free pair of electrons in the empty π* orbital of singlet oxygen. The general reactions taking place could be represented as follows:

^1^O_2_ (^1^Δ_g_) = O_2_ [π* ↑↓   π*] rate determining step—formation of singlet oxygen

$${}^{1}O_{2}\left({}^{1}\Delta_{g}\right)\;+\;\mathrm{antioxidant}\overset{}{\underset{}{⇄}}{\left[\mathrm{adduct}\;\left({}^{1}O_{2}/\mathrm{antioxidant}\right)\right]}^{\neq }\overset{}{\underset{}{⇄}}\mathrm{oxidation}\;\mathrm{products}\;\mathrm{of}\;\mathrm{antioxidants}$$

A similar mechanism has been proposed [1] for the reaction of singlet oxygen with biomolecules: 

^1^O_2_ (^1^Δ_g_) = O_2_ [π* ↑↓   π*] rate determining step—formation of singlet oxygen

$$\begin{array}{l} {}^{1}O_{2}\left({}^{1}\Delta_{g}\right)\;+\;\mathrm{biomolecule}\left(s\right)\;\mathrm{bearing}\;\mathrm{regions}\;\mathrm{of}\;\mathrm{high}\;\mathrm{electron}\;\mathrm{density}\overset{}{\underset{}{⇄}}\\ {}[\mathrm{adduct}{\left({}^{1}O_{2}/\mathrm{biomolecule}\left(s\right)\right]}^{\neq }\overset{}{\underset{}{⇄}}\mathrm{oxidation}\;\mathrm{products}\;\mathrm{of}\;\mathrm{biomolecule}\left(s\right) \end{array}$$

We have presented the regions of high electron density in the chemical structures of various antioxidants. There are not experimental data available for reactions of all of them with oxygen in order to use them for our calculations. Based on their structures, we can suggest that astaxanthin, carotenoids, vitamin K_2_ are very suitable for the reactions with singlet oxygen.

We expect different behaviour of antioxidants in solution experiments (as are the experiments that provided us with the data that we have used for our calculations) and in the cells. We can expect different activation energies for the diffusion of the antioxidants in aqueous solutions and in the cells. E_act_ diffusion in aqueous media < E_act_ diffusion in cells. This explains the smaller ΔG^≠^ that we found in our antioxidants’ calculations. 

There are common features in the structures of the antioxidants i.e., conjugated double bonds, C=O bonds, lone pairs of electrons in O, N, S atoms, etc.

Vitamin C, vitamin E, as well as vitamin K_1_, are not the best antioxidants for the role that we expect them to play since they have very few sites that can overlap with singlet oxygen’s empty π* orbital, whereas astaxanthin, lycopene, beta-carotene etc., have a lot more sites available multiplying by this way their “concentration”.

The competition between proteins, DNA, biomolecules in general and antioxidants for capturing singlet oxygen’s empty π* orbital could be successful for antioxidants by increasing their “useful concentration”.

## Figures and Tables

**Table 1:** Kinetic data (k, T) and calculated thermodynamic parameters (E_act_, ΔH^≠^, ΔS^≠^, ΔG^≠^) for reactions of various antioxidants.

	Τ^(i)^ (Κ)	lnk^(i)^	E_act_^(ii)^ kJmol^−1^	ΔΗ^≠(iii)^ kJ^−1^mol^−1^	ΔS^≠(iii)^ JK^−1^mol^−1^	ΔG^≠(iii)^ kJmol^−1^
Case a Thermal injury due to normal body temperature [23]						
	314	−10.360	769.52^(iii)^	766.89	2110.41	112.8 at 310 K
	315	−9.8338				
	316	−8.4168				
	317	−7.4544				
	318	−6.6079				
	319	−6.0185				
Case b Degradation kinetics of anthocyanins [29] [blackberry juice and concentrate]						
	333	−11.3732	58.90^(iii)^	56.01	−172.01	109.33 at 310 K
	343	−10.7245	58.95^(b)^			
	353	−10.0979				
	363	−9.6309				
Case c Degradation kinetics of anthocyanins [31] [Guatemala roselle]						
	308	−12.0491	39.27^(iii)^	36.46	−226.7	106.75 at 310 K
	340.5	−10.5013				
	371.5	−9.4347				
Case c Degradation kinetics of anthocyanins [31] [BRJ]						
	308	−12.7647	49.17^(iii)^	46.37	−200.31	108.47 at 310 K
	340.5	−10.7615				
	371.5	−9.4962				
Case d Antioxidant activity and carotenoids content [38]						
	323	−14.5426	23.34^(iii)^	20.53	−302.48	114.31 at 310 K
	333	−14.2012	23.7^(d)^			
	353	−13.7892				
Case e Degradation of carotenoid antioxidants [39] (i) cultivar lycopene						
	333	−9.0420	4.62^(iii)^	1.62	−315.66	99.48 at 310 K
	353	−8.8620	4.63^(e)^			
	373	−8.8049				
	393	−8.7829				
(i) cultivar beta-carotene						
	333	−9.0006	4.13^(iii)^	1.13	−317.03	99.41 at 310 K
	353	−8.898	4.14^(e)^			
	373	−8.8274				
	393	−8.7721				
(ii) cultivar lycopene						
	333	−9.5911	8.64^(iii)^	5.64	−307.97	101.11 at 310 K
	353	−9.2616	8.65^(e)^			
	373	−9.2103				
	393	−9.0852				
(ii) cultivar Beta-carotene						
	333	−9.4129	4.81^(iii)^	1.81	−318.17	100.44 at 310 K
	353	−9.2271	4.81^(e)^			
	373	−9.1616				
	393	−9.1458				
Case f Beta-carotene degradation [40]						
	333	−9.6938	95.73^(iii)^	92.88	−46.782	107.37 at 310 K
	343	−8.4143	95.4^(f)^			
	353	−7.7402				
Case g Stability of curcumin during storage [41] With the presence of light						
	278	−8.4068	24.213^(iii)^	21.752	−235.8	94.912 at 310 K
	306	−7.3488	23.88^(g)^			
	318	−7.1247				
						
Case g Stability of curcumin during storage [41] Without the presence of light						
	278	−9.4980	11.803^(iii)^	9.342	−290.3	99.32 at 310 K
	306	−9.3534	13.65^(g)^			
	318	−8.74034				
Case h, Thermal degradation of polyphenols [51] [grape marc]						
	353	−7.1185	5.67^(iii)^	2.45	−298.1	94.86 at 310 K
	373	−6.8831	55.98^(h)^			
	423	−6.7709				
Case h, Thermal degradation of polyphenols [51] [filtered extract]						
	353	−6.7709	9.03^(iii)^	5.80	−285.9	94.46 at 310 K
	373	−6.5583	53.196^(h)^			
	423	−6.2520				
Case i Degradation kinetics of sulforaphane [55]						
pH = 2.2	333	−12.7939	85.36^(iii)^	82.48	−103.66	114.95 at 310 K
	348	−11.1844				
	355	−10.8479				
	363	−10.2289				
pH = 3.0	333	−12.7939	93.17^(iii)^	90.29	−79.97	115.08at310 K
	348	−11.0021				
	355	−10.5966				
	363	−10.0213				
pH = 4	333	−12.1007	79.69^(iii)^	76.80	−114.58	112.32 at 310k
	348	−10.4913				
	355	−10.1548				
	363	−9.7493				
pH = 5	333	−11.4076	71.87^(iii)^	68.98	−132.98	110.2 at 310 K
	348	−10.1548				
	355	−9.7493				
	363	−9.2675				
pH = 6	333	−11.0021	69.61^(iii)^	66.73	−136.01	108.9 at 310 K
	348	−9.6584				
	355	−9.3281				
	363	−8.9437				
Case j Antioxidant activity and thiosulfinate degradation of garlic [80]						
Antioxidant activity	353	−5.5090	89.76^(iii)^	86.74	−46.68	101.21 at 310 K
	363	−4.7736	89.75^(j)^			
	373	−3.8672				
Case j Antioxidant activity and thiosulfinate degradation of garlic [80]						
Degradation of thiosulfinates	353	−5.5684	7.565^(iii)^	4.55	−279.7	91.27 at 310 K
	363	−5.5090	7.67^(j)^			
	373	−5.4299				
Case k, Vitamin C degradation, [91]						
Standard ascorbic acid solution (Merck)	303	−8.7916	36.65^(iii)^	34.09	−205.47	97.79 at 310 K
	313	−8.3266	36.53^(k)^			
Vitamin C 200 mg tablet	303	−7.0845	27.64^(iii)^	25.08	−221.03	93.6 at 310 K
	313	−6.7338	27.82^(k)^			
Ascovit 100 mg tablet	303	−7.1915	11.02^(iii)^	8.46	−276.8	94.26 at 310 K
	313	−7.0516	10.96^(k)^			
Vitamin C nose drops	303	−7.9778	34.89^(iii)^	32.33	−204.53	95.73 at 310 K
	313	−7.5351	35.10^(k)^			
Case l, Temperature and high pressure stability of vitamin C [92] [watermelon]						
	343	−8.4300	76.92^(iii)^	73.98	−101.76	105.528 at 310 K
	353	−8.1404	76.86^(l)^			
	363	−6.9349				
Case l, Temperature and high pressure stability of lycopene [92] [watermelon]						
	343	−11.911	97.21^(iii)^	94.27	−71.56	116.46 at 310 K
	353	−11.423	97.7^(l)^			
	363	−10.024				
Case m, Oxidation of vitamin C [93]						
	313	−12.8992	41.65^(iii)^	38.89	−224.4	108.47 at 310 K
	323	−11.5297	20.73^(m)^			
	333	−11.1765				
	343	−10.9159				
	353	−10.9823				
Case n, Effect of antioxidants [vitamin C, cysteine, fumaric acid] on the stability of an HTK solution [95] HTK						
	323	−13.1563	100.48^(iii)^	97.75	−52.15	113.92 at 310k
	333	−12.032	100.186^(n)^			
HTK + cysteine	323	−13.6903	92.31^(iii)^	89.58	−81.87	114.96 at 310 K
	333	−12.6576	91.96^(n)^			
HTK + vitamin C	323	−13.8494	98.20^(iii)^	95.47	−64.98	115.61 at 310 K
	333	−12.7508	98.127^(n)^			
HTK + fumaric acid	323	−13.6903	89.94^(iii)^	87.21	−89.22	114.87 at 310 K
	333	−12.6841	89.70^(n)^			
Case o, Kinetic parameters of walnut oil [97]						
	353	−9.7921	10.04^(iii)^	6.99	−307.8	102.4 at 310 K
	363	−9.7042	80.32^(o)^	77.27^(o)^	−55.76^(o)^	94.56 at 310 K calculated by us according to the ΔH^≠^, and ΔS^≠^ of work [133]
	373	−9.6146				
	383	−9.5239				
Oxygen-free conditions Case p kinetics and mechanism of diallyl disulfide thermal decomposition [102]						
	433	−9.8302	123.32^(iii)^	119.60	−52.30	135.81 at 310 K
	443	−8.7702				
	453	−8.1101				
	463	−7.5902				

**Notes:** (i) Experimental data in units of T in K and of k in: s^−1^ (cases a, b, c, d, e, f, g, h, i, j, k, l, m, n, o, p) (ii) Calculated values; (iii) This work. **Important Note:** In the cases where our calculated values differ considerably from the values reported in the literature, we noticed that our difference was in the units of the rate constants. The unit of inverse time in k should be in s−1 since the units of the Planck’s constant are in Js, i.e., time is in s (seconds). In some of the cases where our results are different, the unit of inverse time of the k was in min−1 or in h−1 (which is not correct).
